# Notes and Notices

**Published:** 1888-10

**Authors:** 


### NOTES AND NOTICES.

Removals.—Dr. R. B. Johnstone, from 1429 Chestnut Street to 1319 Walnut Street, Philadelphia; Dr. G. W. Sherbino, from Abilene to Dallas, Texas; Dr. Jennie V. H. Baker, from 245 South First Street to 512 Bedford Avenue, Brooklyn; Dr. W. Steinrauf, from Nokomis, Illinois, to St. Charles, Missouri.

